# Chemical kinetics of silver diammine fluoride in demineralization and remineralization solutions—an *in vitro* study

**DOI:** 10.3389/froh.2024.1374333

**Published:** 2024-04-19

**Authors:** M. Kaur, P. Anderson, S. Shahid, F. S. L. Wong

**Affiliations:** Dental Physical Sciences Unit, Centre for Oral Bioengineering, Institute of Dentistry, Faculty of Medicine and Dentistry, Queen Mary University of London, London, United Kingdom

**Keywords:** SDF, demineralization, remineralization, XRD, NMR, AgCl

## Abstract

**Introduction:**

Silver Diammine Fluoride (SDF) is a clinical minimal intervention to manage dentin caries. Its chemistry in demineralization conditions has been investigated widely, but far less in remineralization conditions. The aim was to investigate and compare the chemical reactions when SDF is added to remineralization and demineralization solutions.

**Methods:**

0.01 ml SDF (Riva Star) was added to deionized water (DW); demineralization (DS = pH4) and remineralization (RS = pH7.0) solutions. The time sequence of concentrations of NH_4_^+^, F^−^, and Ag^+^ were measured using ion selective electrodes (ISEs) every 2 min. The pH was also measured. Precipitates were characterized using x-ray Diffraction (XRD) and, ^31^P and ^19^F nuclear magnetic resonance spectroscopy (NMR).

**Results:**

The concentrations of NH_4_^+^ and Ag^+^ showed decreasing trends in DW (−0.12 and −0.08 mM/h respectively), and in DS (−1.06 and −0.5 mM/h respectively); with corresponding increase in F^−^ concentration (0.04 and 0.7 mM/h respectively). However, in RS, NH_4_^+^ concentration showed little change (0.001 mM/h), and Ag^+^ and F^−^ concentrations were negligible. XRD results showed that precipitates (in RS only) contained AgCl, and metallic Ag. NMR showed that fluorapatite/carbonated fluorapatite (FAP/CFAP) were formed. The pH increased after SDF addition in all three solutions.

**Discussion:**

SDF dissolved to release NH_4_^+^, F^−^ and Ag ^+ ^. In DW and DS, NH_4_^+^ combined with Ag^+^ to form diamminesilver, causing an increase of F^−^ and pH. In RS, F^−^ reacted with Ca^2+^ and (PO)_4_^3−^ to form FAP/CFAP, and Ag^+^ reacted with Cl^−^ to form AgCl/Ag. These suggests why SDF is effective in managing dentin caries.

## Introduction

Demineralization and remineralization studies serve as crucial tools in investigating the efficacy of therapeutic interventions on dental substrates such as hydroxyapatite (HA) discs, enamel, or dentin. These investigations simulate the cariogenic challenges encountered in the oral cavity and provide valuable insights into potential treatments for caries prevention and management.

While various models have been employed in cariology research to replicate the caries process, chemical models offer distinct advantages in terms of efficiency, cost-effectiveness, and reproducibility. Among these models, chemical approaches utilizing acid or acid buffers to mimic demineralization and remineralization mechanisms have gained prominence. Notably, Yu et al. (1) observed that 62% of mechanistic studies utilize simple mineralization models, while 38% employ pH cycling models.

Silver diammine fluoride (SDF) is a colorless liquid and is a promising agent for dentin caries management. Its application involves painting it onto carious lesions for a brief duration. Therefore, understanding the chemical interaction of SDF in both acidic and remineralization environments is important.

Ion-selective electrodes (ISEs) are an analytical tool for determining the concentration of specific free ions in solution. In dental research, ISEs have a proven efficacy in various studies. For example, Huang et al. (2) demonstrated that calcium ISEs is a reliable method for real-time quantification of mineral loss during demineralization using a hydroxyapatite model system.

While numerous investigations have explored the effect of SDF on HA during demineralization, but there are noticeably fewer investigating its impact during remineralization (3). The aim of this study was to compare the chemical kinetics of SDF following dissolution in water, demineralization solutions, and in remineralization solutions. The emphasis was to investigate the chemical reactions in real-time of following addition of SDF into de- and re-mineralization solutions rather than its reactions directly on tooth tissues.

## Materials and methods

Commercially available SDF (Ag(NH_4_)_2_F) (Riva Star, SDI, Australia) was used which has a concentration of 3.16M. Demineralization solutions of buffered 0.1M acetic acid at pH4.0 were prepared. Remineralization solutions comprising of 0.222 g/L CaCl_2_, 0.163 g/L KH_2_PO_4_, 8.7 g/L NaCl at pH7 were also prepared (4). 0.01 ml of SDF was added to 50 ml of: deionized water, demineralization solution, and remineralization solutions, thus resulting in a concentration of 0.632 mM of SDF in each. ISEs (Nico2000, UK) were used to measure initial, and also to continually monitor NH_4_^+^_,_ Ag^+^ and F^−^ concentrations at intervals of 2 min (2) for a period of 2 h. All the experiments were carried out at 37 °C (repeated three times). The pH of the solution was measured before addition of SDF, and at the end of the experiment using a calibrated pH meter (Mettler Toledo portable pH meter).

Any precipitates were collected and dried in an incubator and characterized using x-ray diffraction (XRD), and ^31^P and ^19^F solid state Magic Angle Spinning nuclear magnetic resonance (MAS-NMR) spectroscopy. XRD spectra were collected using a x-ray powder diffractometer (PANalytical CubiX^3^, UK). The theta-2theta scan measurements were carried out in standard reflection mode, using Cu K radiation, with sample holders spinning on the stage during the scan. ^31^P MAS-NMR was conducted using a 14.1 Tesla spectrometer (600 MHz Bruker, UK) at a Larmor frequency of 242.94 MHz. ^19^F MAS NMR analysis was conducted using a 14.1 Tesla spectrometer at a Larmor frequency of 564.658 MHz. All spectra were obtained with a 2.5 mm probe under spinning conditions of 20 kHz.

## Results

**Figure 1:**
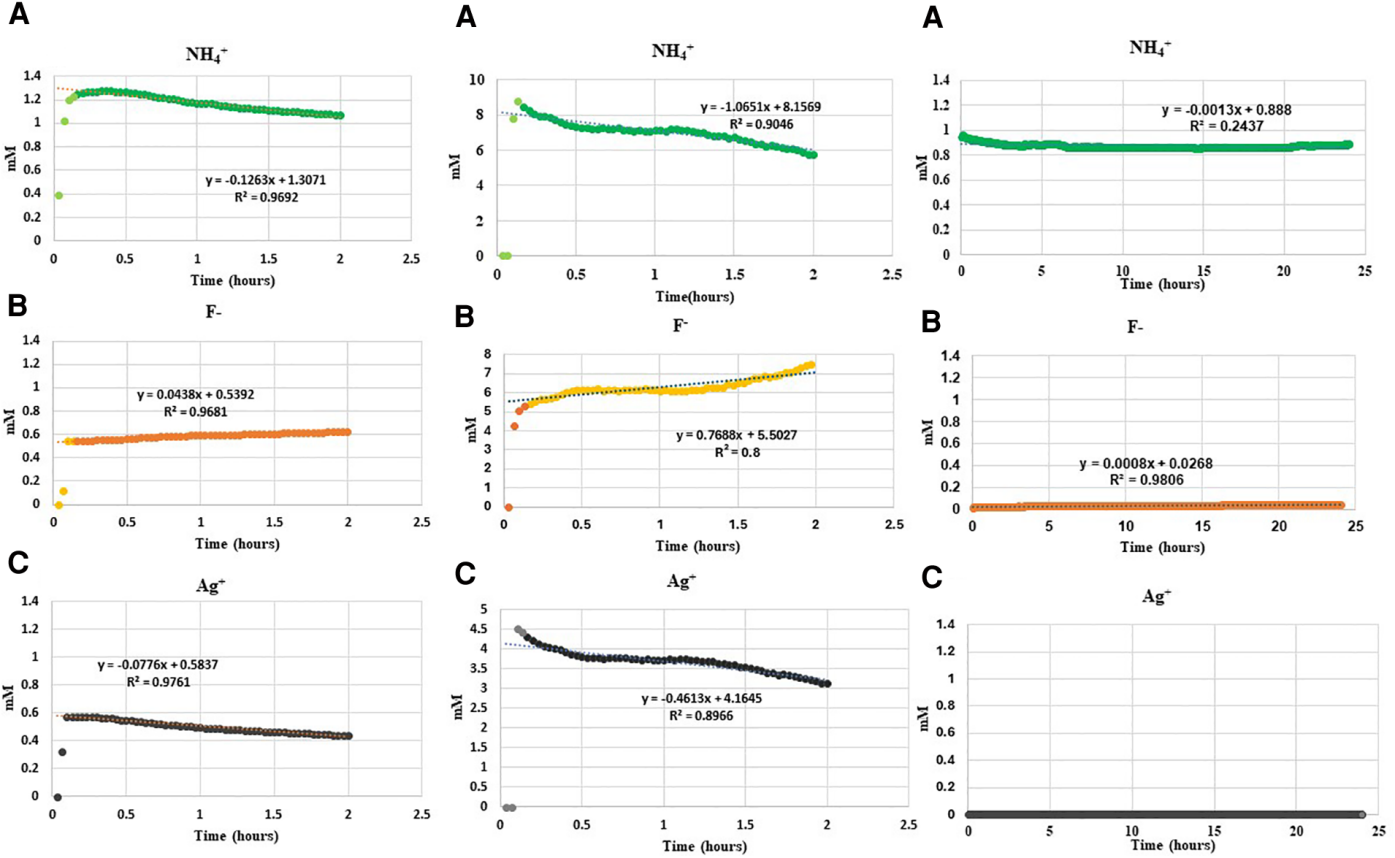
Changes in concentration of (**A**) NH_4_^+^, (**B**) F^−^ and (**C**) Ag^+^ ions on addition of 0.01 ml SDF (3.16 M) to 50 ml deionized water, demineralization solution at pH4, and remineralization solution at pH7 in a time series. *Please note that the concentration of Ag^+^ ions in the remineralization solution was below the lowest detection limit of ISE as a result of immediate precipitation.

The ISE results of the real-time changes of free NH_4_^+^, F^−^ and Ag^+^ in the water, demineralization and remineralization solutions are shown in Figure 1. The trends after initial values were similar in deionized water and the demineralization solutions, i.e., decreasing concentration with time for NH_4_^+^, and Ag^+^, but increasing for F^−^. Whereas, for remineralization solution, after initial solubilization, following addition of SDF, the trend was no change in NH_4_^+^ and very low with virtually no change in Ag^+^ and F^−^ concentrations.

### Ammonium ions

Following addition of SDF into deionized water, the initial NH_4_^+^ concentration increased immediately to 1.2 mM, and then subsequently decreased linearly by 0.12 mM/h. Following addition of SDF into demineralization solution the NH_4_^+^ concentration increased rapidly to 9.0 mM, and then subsequently decreased linearly by 1.06 mM/h. However, following addition of SDF into remineralization solution, the initial NH_4_^+^ concentration increased to 1.0 mM but subsequently did not change much over time.

### Fluoride ions

The initial F^−^ concentration in deionized water was 0.6 mM on addition of SDF, and then increased by 0.04 mM/h. The initial F^−^ in demineralizing solution was 6 mM on addition of SDF, and then increased by 0.7 mM/h. In the remineralization solution, the F^−^ concentration was between 0.025 and 0.045 mM on addition of SDF, and did not change subsequently.

### Silver ions

The initial Ag^+^ concentration in deionized water was 0.6 mM on addition SDF, and then decreased by 0.08 mM/h. Following addition of SDF in demineralization solution, the concentration of Ag^+^ was 4.5 mM, and then decreased by 0.5 mM/h. Whereas, following addition of SDF to the remineralization solution, the Ag^+^ concentration did not rise above the minimum detection limit of the ISE electrode.

#### Precipitation of solids

**Figure 2:**
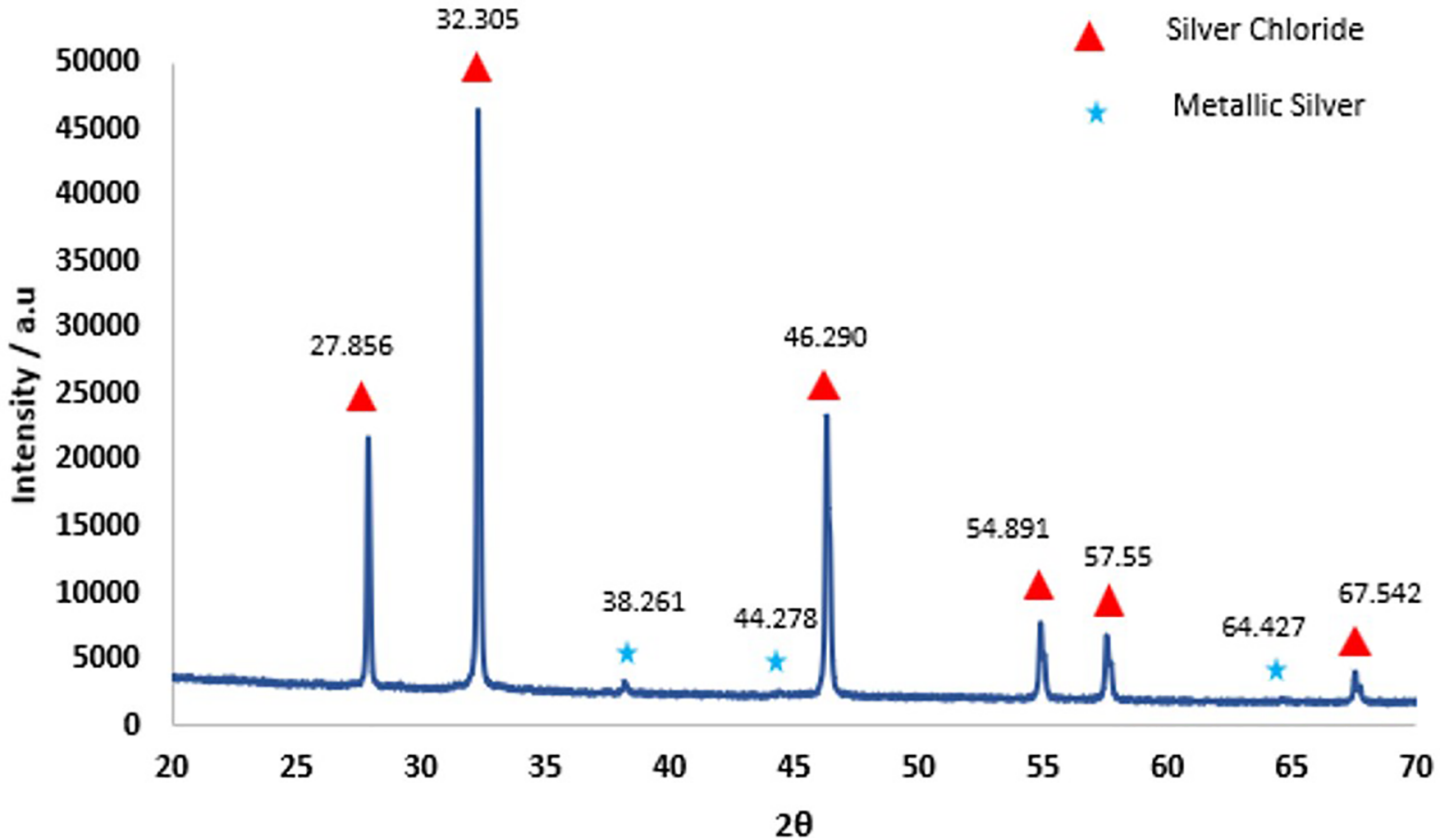
X-ray diffraction results obtained from the precipitate formed as a result of interaction of SDF with remineralization solution. The sharp diffraction lines and the pattern seen above indicate AgCl crystal (Reference Code: 04-007-3906) and small amount of metallic silver (Reference code: 04-006-1881).

**Figure 3:**
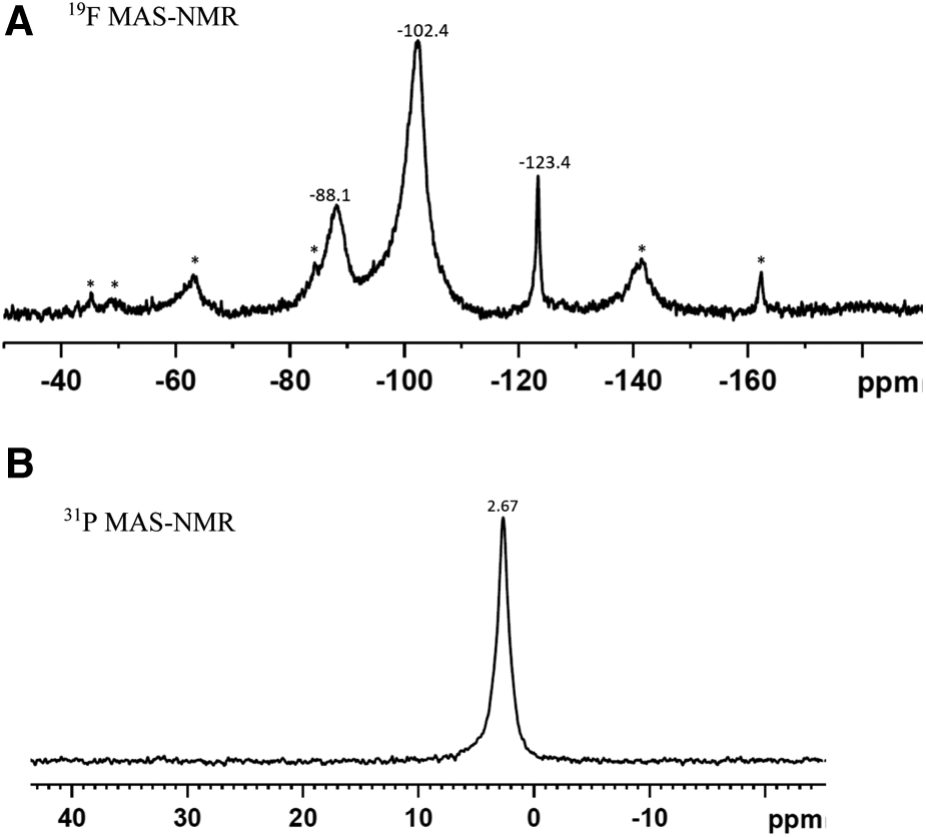
(**A**) ^19^F MAS-NMR, and (**B**) ^31^P MAS-NMR spectra of the precipitate formed as a result of interaction of SDF with remineralisation solution. The Asterix denote the sidebands. (**A**) The peak at −102.4 ppm indicates the formation of fluorapatite and at −88.1 ppm, carbonated complex. (**B**) The peak at 2.67 ppm signifies formation of apatite.

No precipitate was seen when SDF was added to either deionized water or demineralization solution. However, when SDF was added to remineralization solution, there was a rapid change in color from clear to white and a white precipitate was formed. XRD pattern of the precipitate (Figure 2) showed peaks at 27.9°, 32.3°, 46.3°, 54.9° and 57.6° that could be assigned to silver chloride (AgCl, Reference code-04-007-3906). The small peaks at 38.3°, 44.3° and 64.4° are consistent with the formation of metallic silver (Reference code: 04-006-1881). The ^19^F MAS-NMR spectrum (Figure 3A) shows a peak at −102 ppm suggesting the formation of fluorapatite (FAP); and the peak at −88 ppm suggesting the formation of carbonated fluorapatite (CFAP) (5, 6). The ^31^P MAS-NMR spectrum peak at 2.7 ppm indicates the presence of calcium hydroxyapatite (Figure 3B).

### pH

The change of pH in the solutions is presented in Table 1. The pH increased after the addition of SDF in all solutions. The greatest increase in pH was in deionized water and the least was in demineralization solution.

**Table 1:** Change of pH in solutions on interaction with SDF before and after experimental period.

Change in pH	Deionised water	Demineralisation solution	Remineralisation solution
Before SDF	7.7	4.0	7.0
After SDF	10.3	4.4	8.4

## Discussion

The complete dissolution of SDF in any of the solutions would result in concentrations of 1.2 mM NH_4_^+^, 0.6 mM F^−^, and 0.6 mM Ag^+^ calculated from the stoichiometry and concentration and volumes of SDF used, and assuming complete dissociation. SDF is known to be soluble in water (7). The ISE results in deionized water showed that it completely dissociated to give rise to around 1.2 mM NH_4_^+^_,_ and 0.6 mM Ag^+^ and F^−^ ions after adding 0.01 ml of 3.16 mM SDF in 50 ml of water. The subsequent small decrease of NH_4_^+^ and Ag^+^ in the following 2 h indicated that an aqueous ammonia-silver ion complex, likely to be diamminesilver [Ag(NH)_3_)_2_^+^] (8, 9), leading to a corresponding increase in F^−^ in the solution, which might be the cause for the pH increase.

This situation was similar for SDF dissolved in demineralization solution as the gradients of the decrease of NH_4_^+^ and Ag^+^ concentrations, and the corresponding gradient of increase for F^−^, were similar to that in deionized water. As Ag(NH)_3_)_2_^+^ is a stable aqueous species, and its ammonia-silver covalent bonds prevent the photo-reduction of silver, no precipitate was formed and the solution remained clear.

However, when SDF was dissolved in remineralization solution, the NH_4_^+^ concentration remained constant at about the stoichiometric value, but the concentrations of F^−^ and Ag^+^ were both very low. This indicated that these ions were taken up immediately to form the precipitate. The XRD and NMR analyses (Figures 2, 3) showed that these precipitates contained a mixture of AgCl, metallic silver, and fluorapatites, which are all insoluble compounds. As the remineralization solution contained CaCl_2_, KH_2_PO_4,_ and NaCl, this suggests that the dissociated Ag^+^ reacted rapidly with the Cl^−^ to form AgCl as a white precipitate, thus reducing the Ag^+^ concentration in the solution to a negligible amount. Subsequently, when the AgCl precipitate was exposed to light, a small amount was then photo-reduced to metallic silver, which was also seen in the XRD analysis. The F^−^ would react immediately with Ca^2+^ to form CaF_2_ (10, 11), another white precipitate, which in the presence of phosphate, would form insoluble fluorapatite, (and therefore was not seen in the NMR spectra). Thus, the F^−^ concentration was also very little (Figure 1C).

This *in vitro* study, investigating the kinetics of the chemical reactions of SDF in demineralization and remineralization conditions can be used to predict the effects of SDF in the oral environment. As saliva contains Ca^2+^, (PO_4_)^3­−^ and Cl^−^, these ions would also react with SDF to form insoluble FAP/CFAP and AgCl. These precipitates would then be retained for example in exposed dentinal tubules and not be washed away. The formation of the FAP would render the dentin to be less susceptible to acid demineralization (12–14). AgCl is photo-reduced to metallic Ag (giving the black appearance seen when SDF is used in dentin treatments) which will hinder bacterial growth and degradation of dentin collagen (15, 16). As the components of SDF also increase the pH in the oral environment, this will further reduce the damage caused by acid challenges. Furthermore, as the AgCl and FAP/CFAP precipitate within the dental tubules, these precipitated compounds may occlude the tubular lumens and minimize pain by reducing direct communication of the pulp with external stimuli. However, in enamel, these precipitates could only be retained in demineralized porous lesions, and not on sound smooth surface. Therefore, clinically, the SDF protective benefit against acidic challenge for enamel (17) is reduced compared to dentin (13). Other studies [e.g., (14)] also demonstrated that the use of SDF/NaF had a remineralizing effect on a dentin surface under acid challenge.

Clearly, this *in vitro* study is limited if compared to the complexity of the *in vivo* oral environment, where there are a multitude of other charged species (ions and proteins) in saliva, all of which may partially interact with the ionic constituents of SDF. In the oral environment both demineralization and remineralization occur within the complex biological fluid that is saliva (18). Enamel biodemineralization (19, 20) has been proposed as the process by which tooth mineral is lost within a chemically interacting environment. Nevertheless, this *in vitro* study does demonstrate that complexes are formed when SDF is dissolved, especially when other ions including Cl^−^ are present, which is a major component of saliva.

## Conclusion

In remineralizing solutions which contain chloride ions (and therefore likely to be the case in saliva), SDF reacts rapidly with calcium, phosphate, and chloride ions, leading to the formation of fluorapatite, carbonated fluorapatite, and silver chloride precipitates. The silver chloride precipitate will undergo photo-reduction, resulting in black discoloration. These precipitates, which would be retained inside dentinal tubes, with the increases of pH, suggests why SDF is beneficial in clinical treatments for dentin caries.

## Data Availability

The original contributions presented in the study are included in the article/Supplementary Material, further inquiries can be directed to the corresponding author.

## References

[1] Yu OY Zhao IS Mei ML Lo EC-M Chu C-H. A review of the common models used in mechanistic studies on demineralization-remineralization for cariology research. Dent J. (2017) 5(2):20. 10.3390/dj5020020 PMC5806972 29563426

[2] Huang WT Shahid S Anderson P. Validation of a real-time ISE methodology to quantify the influence of inhibitors of demineralization kinetics in vitro using a hydroxyapatite model system. Caries Res. (2018) 52(6):598–603. 10.1159/000488597 29804111

[3] Kaur M Shahid S Karpukhina N Anderson P Wong FSL. Characterization of chemical reactions of silver diammine fluoride and hydroxyapatite under remineralization conditions. Front Oral Health. (2024) 5(March). 10.3389/froh.2024.1332298 38496333 PMC10940413

[4] Siddiqui S Anderson P Al-Jawad M. Recovery of crystallographic texture in remineralized dental enamel. PLoS One. (2014) 9(10):e108879. 10.1371/journal.pone.0108879 25360532 PMC4215832

[5] Yi H Balan E Gervais C Segalen L Fayon F Roche D Letter. A carbonate-fluoride defect model for carbonate-rich fluorapatite. Am Mineral. (2013) 98(5–6):1066–9. 10.2138/am.2013.4445

[6] Hiraishi N Sayed M Hill RG Shimada Y. Solid-state NMR spectroscopy measurement of fluoride reaction by bovine enamel and dentin treated with silver diammine fluoride. Dent Mater. (2022) 38(5):769–77. 10.1016/j.dental.2022.04.017 35450704

[7] Jackson JK Dietrich C Shademani A Manso A. The manufacture and characterization of silver diammine fluoride and silver salt crosslinked nanocrystalline cellulose films as novel antibacterial materials. Gels. (2021) 7(3):104. 10.3390/gels7030104 34449599 PMC8395774

[8] Chu C Lo ECM. Promoting caries arrest in children with silver diamine fluoride: a review. Oral Health Prev Dent. (2008) 6(4):315–21. 10.3290/j.ohpd.a14177 19178097

[9] Jiang M Mei ML Wong MCM Chu CH Lo ECM. Influence of silver diamine fluoride treatment on the microtensile bond strength of glass ionomer cement to sound and carious dentin. Oper Dent. (2020) 45(5):E271–9. 10.2341/19-237-l 32502257

[10] Mohammed NR Kent NW Lynch RJ Karpukhina N Hill R Anderson P. Effects of fluoride on in vitro enamel demineralization analyzed by ^19^F MAS-NMR. Caries Res. (2013) 47(5):421–28. 10.1159/000350171 23712030

[11] Ferizoli B Cresswell-Boyes AJ Anderson P Lynch RJM Hill RG. Effects of fluoride on in vitro hydroxyapatite demineralisation analysed by ^19^F MAS-NMR. Front Dent Med. (2023) 4(May). 10.3389/fdmed.2023.1171827 PMC11797780 39916921

[12] Mei ML Nudelman F Marzec B Walker JM Lo ECM Walls AW Formation of fluorohydroxyapatite with silver diamine fluoride. J Dent Res. (2017) 96(10):1122–8. 10.1177/0022034517709738 28521107 PMC5582683

[13] Yu OY Zhao IS Mei ML Lo EC Chu CH. Caries-arresting effects of silver diamine fluoride and sodium fluoride on dentine caries lesions. J Dent. (2018b) 78:65–71. 10.1016/j.jdent.2018.08.007 30114443

[14] Cifuentes-Jiménez CC Bolaños-Carmona MV Enrich-Essvein T González-López S Álvarez-Lloret P. Evaluation of the remineralizing capacity of silver diamine fluoride on demineralized dentin under pH-cycling conditions. J Appl Oral Sci. (2023) 31:e202203. 10.1590/1678-7757-2022-0306 PMC10065761 36995879

[15] Mei ML Chu CH Low KH Che CM Lo EC. Caries arresting effect of silver diamine fluoride on dentine carious lesion with *S. mutans* and *L. acidophilus* dual-species cariogenic biofilm. Med Oral Patol Oral Cir Bucal. (2013) 18:e824–31. 10.4317/medoral.18831 23722131 PMC3854072

[16] Mei ML Lo EC. ‘Arresting dentine caries with silver diamine fluoride: what’s behind it? J Dent Res. (2018) 97(7):751–8. 10.1177/0022034518774783 29768975

[17] Yu OY Mei ML Zhao IS Li Q Lo EC Chu CH. Remineralisation of enamel with silver diamine fluoride and sodium fluoride. Dent Mater. (2018a) 34(12):e344–52. 10.1016/j.dental.2018.10.007 30482611

[18] DePaola DP. Saliva. J Am Dent Assoc. (2008) 139(May):5S–6S. 10.14219/jada.archive.2008.0348 18460673

[19] Wang L Nancollas GH. Pathways to biomineralization and biodemineralization of calcium phosphates: the thermodynamic and kinetic controls. Dalton Trans. (2009) 15(January):2665. 10.1039/b815887h 19333487

[20] Al-Jawad M Anderson P. Biomineralization and biodemineralization of enamel. In: Matinlinna JP, editor. Handbook of Oral Biomaterials. 1st ed. United Kingdom: Jenny Stanford Publishing (2014). p. 25–80. 10.1201/B15644-3

